# Genetic mapping of legume orthologs reveals high conservation of synteny between lentil species and the sequenced genomes of *Medicago* and chickpea

**DOI:** 10.3389/fpls.2014.00676

**Published:** 2014-12-05

**Authors:** Neha Gujaria-Verma, Sally L. Vail, Noelia Carrasquilla-Garcia, R. Varma Penmetsa, Douglas R. Cook, Andrew D. Farmer, Albert Vandenberg, Kirstin E. Bett

**Affiliations:** ^1^Department of Plant Sciences, University of SaskatchewanSaskatoon, SK, Canada; ^2^Department of Plant Pathology, University of California, DavisDavis, CA, USA; ^3^Bioinformatics, National Centre for Genomic ResourcesSanta Fe, NM, USA

**Keywords:** wild lentil, legume tentative orthologs, mapping, translocation, synteny, *Medicago*

## Abstract

Lentil *(Lens culinaris* Medik.) is a global food crop with increasing importance for food security in south Asia and other regions. *Lens ervoides*, a wild relative of cultivated lentil, is an important source of agronomic trait variation. *Lens* is a member of the galegoid clade of the Papilionoideae family, which includes other important dietary legumes such as chickpea (*Cicer arietinum*) and pea (*Pisum sativum*), and the sequenced model legume *Medicago truncatula*. Understanding the genetic structure of *Lens* spp. in relation to more fully sequenced legumes would allow leveraging of genomic resources. A set of 1107 TOG-based amplicons were identified in *L. ervoides* and a subset thereof used to design SNP markers for mapping. A map of *L. ervoides* consisting of 377 SNP markers spread across seven linkage groups was developed using a GoldenGate genotyping array and single SNP marker assays. Comparison with maps of *M. truncatula* and *L. culinaris* documented considerable shared synteny and led to the identification of a few major translocations and a major inversion that distinguish *Lens* from *M. truncatula*, as well as a translocation that distinguishes *L. culinaris* from *L. ervoides*. The identification of chromosome-level differences among Lens spp. will aid in the understanding of introgression of genes from *L. ervoides* into cultivated *L. culinaris*, furthering genetic research and breeding applications in lentil.

## Introduction

Lentil (*Lens culinaris* Medik.; Lc) is the fifth most important grain legume crop globally and an important export crop for Canada. It is cultivated primarily in North America, Australia, the Middle East, Western Asia, and the Indian subcontinent (Erskine, 1996). It is self-pollinating with a diploid genome (2n = 14) of 4 Gbp (Arumuganathan and Earle, 1991). Similar to other legume crops, lentil fixes atmospheric nitrogen and provides rotational and environmental benefits, such as management of insect pests, diseases, and weeds. It is a valuable cash crop and provides an important source of dietary protein, carbohydrates, and vitamins for the human diet. The small seeds cook quickly, giving this crop a culinary advantage over other grain legumes.

Narrow genetic diversity limits genetic improvement of lentil, while susceptibility to diseases and weed infestations are the main production constraints. Wild relatives are valuable sources of resistance genes for crop species, and introgression of these genes plays an important role in increasing genetic variation (Zhao et al., 2010; Cornille et al., 2012; Hufford et al., 2013). The secondary gene pool of cultivated lentil, especially *L. ervoides* (Le), is a source of resistance to anthracnose races Ct0 and Ct1 (Tullu et al., 2006; Fiala et al., 2009; Vail et al., 2011), stemphylium blight (Podder et al., 2013), and ascochyta blight (Bayaa et al., 1994). Inter-specific hybrids derived from a cross between Lc and Le show significant resistance to anthracnose, likely derived from genes present in the wild parent (Tullu et al., 2006).

The development of molecular markers has accelerated trait mapping studies in legumes (Gupta and Varshney, 2000; Andersen and Lübberstedt, 2003; Cannon et al., 2009; Varshney et al., 2009), and gene-based molecular markers are emerging as important tools for molecular mapping in lentil (Hamwieh et al., 2005; Alo et al., 2011; Kaur et al., 2011; Fedoruk et al., 2013). Single-nucleotide polymorphism (SNP)-based markers are ideal for diversity assessment and genetic mapping in crop species due to their high abundance, relatively even distribution across the genome, and the virtual absence of homeoplasy (Varshney, 2010; Gujaria et al., 2011; Sharpe et al., 2013). Lentil has lagged in genomic resource development because of its minor status relative to large global grain crops and its large genome size, though recent efforts by several groups have led to an increase in availability of sequence datasets (Kaur et al., 2011; Sharpe et al., 2013). Rapid advances in massively parallel sequencing and genotyping have revolutionized genomics by reducing both its cost and time by several orders of magnitude (Mardis, 2008; Metzker, 2009; Varshney, 2010). Among the high-throughput genotyping platforms available, the GoldenGate assay from Illumina Inc. (San Diego, CA) has proven to be efficient and useful for genetic fingerprinting (Hyten et al., 2010; Yan et al., 2010; Zhao et al., 2010; Blair et al., 2013). GoldenGate assays have been developed for several legume crops, including common bean (Blair et al., 2013), soybean (Hyten et al., 2008), cowpea (Muchero et al., 2009), pea (Deulvot et al., 2010), chickpea (Gaur et al., 2012), and most recently, lentil (Sharpe et al., 2013).

Lentil belongs to the galegoid sub-family of the Papilionoideae and is a member of the Vicieae tribe. Vicieae consists of the important crop and model legumes *Medicago truncatula* (Mt), lentil, field pea (*Pisum sativum* L.) and faba bean (*Vicia faba* L.). Most of the genes in papilionoid legume species are likely to be found within syntenic regions ranging in size from hundreds of kilobases to several megabases with respect to any other given papilionoid species (Cannon et al., 2009). If a gene and phenotype are experimentally associated in one species, then an orthologous gene is likely to be found in a similar genomic region in closely related species. Taking advantage of this makes comparative genetic mapping a powerful tool for establishing shared ancestry between species and leveraging information from more well-studied species. Genomic relationships have been charted between pea and lentil (Weeden et al., 1992), Mt and pea (Choi et al., 2004a,b; Kalo et al., 2004), and Lc and Mt (Phan et al., 2006; Alo et al., 2011; Sharpe et al., 2013).

Conserved orthologous sequences (COS) have been used to derive markers from a set of low-copy orthologous genes (tentative orthologous genes; TOGs) present in a legume expressed sequence tag (EST) dataset of Mt, lotus (*Lotus japonica*) and soybean (*Glycine max*) transcripts (Penmetsa et al., unpublished). The fact that TOGs have retained their mostly single-copy nature during speciation, they can be helpful in the study of genome structure and evolution across species (Fulton et al., 2002; Choi et al., 2006). Legume TOGs have been exploited to trace the evolutionary history of pigeonpea (*Cajanus cajan*) (Kassa et al., 2012) and common bean *(Phaseolus vulgaris*) (Blair et al., 2013). Mapping of these low copy number sequences in chickpea (Hiremath et al., 2012) and pigeonpea (Saxena et al., 2012) has also helped in the comparison of their genome structures with those of Mt, lotus and soybean (Cook et al., unpublished). Conserved synteny among legume species is often punctuated by chromosomal rearrangements, reflected by differences in chromosome numbers among their genomes. These chromosome rearrangements can be explained by translocations or inversions within legume species (Choi et al., 2004a). Ladizinsky et al. (1984) concluded that *L. nigricans* and *L. lamottei* differ by four reciprocal translocations and one paracentric inversion, resulting in the complete sterility of hybrids with Lc. Ladizinsky et al. (1985) and Tadmor et al. (1987) used crosses between Lc and Le to conclude that a single reciprocal translocation differentiates the two species. Weeden et al. (1992) used one of those interspecific populations and isozyme markers to map the translocation region.

This study was undertaken to capitalize on the recent development of genomic resources in lentil, with objectives to: (i) sequence intron spanning amplicons from the legume TOGs in lentil, (ii) generate TOG-based SNP genotyping assays, (iii) develop a SNP-based genetic map of *L. ervoides*, and (iv) determine macrosyntenic relationships between wild and cultivated lentil and with those of closely related galegoid legumes.

## Materials and methods

### DNA amplification and sequencing

Le accessions IG 72815 (from Turkey) and L01-827a (a single plant selection from IG 72847; Fiala et al., 2009), and Lc cultivars “CDC Redberry” (Vandenberg et al., 2006) and “Eston” (Slinkard, 1981) were selected for TOG marker development. DNA was extracted from freeze-dried leaf tissue of 2-week-old seedlings by a modified CTAB method (Doyle and Doyle, 1990). The quality and quantity of DNA were assessed using 1% agarose gel and a FLUOstar Omega flourimeter (BMG Labtech). DNA was normalized to 50 ng/μL for sequencing and genotyping.

LR-66 is a population developed from a cross between the two Le accessions. An F_2_ population of 91 individuals was generated. LR-59 is an inter-species cross derived from a cross between Eston (Lc) and L01-827a (Le). A F_7:8_ RIL population of 68 individuals had already been developed (Fiala et al., 2009). DNA for LR-66 and LR-59 was extracted and quantified as described above for the Le accessions and Lc cultivars.

### Sanger sequencing and SNP identification

PCR amplicons for Sanger sequencing were generated using primers designed to amplify 1440 TOGs (Penmetsa et al., unpublished) using Le genotypes IG 72815 and L01-827a, and Lc genotypes CDC Redberry and Eston as DNA templates. Sequencing from both 3′- and 5′-ends using forward and reverse primers was carried out and the resulting forward and reverse read amplicon sequences were first assembled for each genotype using CAP3 (Huang and Madan, 1999). The resulting contigs along with any unassembled reads were included in another round of assembly by CAP3 to produce a master alignment from which SNPs were identified using an in-house bioinformatics pipeline at the National Center for Genome Resources (NCGR; Santa Fe, NM). All SNPs were manually curated to verify the alleles called by the software. All contigs and identified SNPs are available through the KnowPulse webportal at http://knowpulse2.usask.ca/portal/node/53 and in Additional file 1b.

### SNP genotyping

#### Illumina GoldenGate assay

To design an Illumina GoldenGate Oligonucleotide Pool Assay (OPA), a set of 9575 validated SNPs was submitted to Illumina for Assay Design Tool (ADT) scoring. Only SNPs with an ADT score of 0.5 or higher were considered for OPA design. SNPs were examined for informativeness based on anticipated polymorphism between Lc and Le or Le and Le mapping populations, and to maximize the number of TOGs covered. A set of 768 SNPs were selected from this filtering and sent to Illumina for the synthesis of the Le768 SNP OPA assay.

The 91 F_2_ individuals from the LR-66 population and 68 LR-59 RILs were genotyped using this Le768 OPA and the standard Illumina GoldenGate Assay protocols (http://www.illumina.com/technology/goldengate_genotyping_assay.ilmn). The products generated by this assay were read with an Illumina iScan (Illumina Inc., San Diego, CA). The resulting data were clustered for allele calling using GenomeStudio software version 2010.3 (Illumina, San Diego, CA). Allele calls were manually checked for errors and corrected as necessary or scored as missing data to exclude their further use. To avoid additional technical errors, all SNPs showing unexpected parental alleles or high levels of missing data (>10%) were excluded from further analysis.

#### Single SNP assays

An additional 31 gene-based SNPs, polymorphic between L01-827A and IG 72815, were identified through a separate project (Sharpe et al., 2013). Allele-specific forward primers and a common reverse primer were designed for use in KASP™ (Kompetitive Allele Specific PCR) assays (LGC Genomics Ltd. Hoddesdon, U.K.; http://www.lgcgenomics.com/kasp-genotyping-reagents). Assays were carried out in a 10 μL reaction volume consisting of 50 ng/μL DNA, KASP 1X Reaction Mix, and 0.17 μM KASP Assay Mix (allele-specific primers and common primer). PCR amplification was carried out in StepOnePlus™ Real-Time PCR System (Applied Biosystems) and fluorescence was analyzed using StepOne Software version 2.1 (Applied Biosystems). Allele calling was done graphically as scatter plots for each marker using SNPViewer (LGC Genomics Ltd., Hoddesdon, UK).

A total of 110 TOG-based SNPs were identified between the two Lc lines for which there was sufficient flanking sequence to design allele-specific primers for KASP assays. Sequence information was sent to KBioscience (now LGC Genomics Ltd.) for assay design and genotyping on the parents of mapping populations, including LR-66.

### Genetic mapping

LR-66 (Le × Le) F_2_ individuals were genotyped using the Le768 OPA and KASP assays. Polymorphic loci were used for mapping using the regression mapping function in JoinMap 4.0 (Van Ooijen, 2006) with a minimum LOD value of 5.0. A Kosambi mapping function was used to convert recombination frequency into centimorgan (cM) distances. Linkage maps were drawn with MapChart version 2.1 (Voorrips, 2002).

### Comparative mapping with closely related legumes

A comprehensive genetic map of Lc based on the mapping population LR-18 (Lc × Lc) developed by Fedoruk et al. (2013) was compared with the Le map (LR-66) developed in this study. Due to a lack of common markers, it was necessary to align both to the *Medicago* genome (version 3.5.1) to facilitate the comparison. Homologs between Le and Mt as well as Lc and Mt were identified using the NUCmer pipeline of the MUMmer software (Kurtz et al., 2004). The visual comparison of these two maps with Mt was made using Strudel, a freely available software package (Bayer et al., 2011; http://bioinf.hutton.ac.uk/strudel/).

Genotypic data for the interspecies RIL population, LR-59 (Le × Lc; data not shown), were used to further examine putative translocations. Common markers for Le linkage groups (LG) LeLG1, LeLG4, LeLG5, and LeLG7 were selected and their order across individuals the Le × Lc inter-specific population LR-59 examined visually.

The orthologous map positions of TOGs mapped in Le were extracted from the Legume Information System (LIS; http://cmap.comparative-legumes.org) for chickpea and Mt. Map units for lentil and chickpea were converted to base pair distances to facilitate comparisons with Mt at an assumed factor of 250,000 bp per cM. Inter-genomic comparisons were drawn using Circos, which is a Perl script based visualization tool to facilitate the identification and analysis of synteny and differences between the genomes arising from comparative analysis (Krzywinski et al., 2009; http://circos.ca/). For a better comparison of the translocation on Lc (Sharpe et al., 2013) with respect to Le, dotplots were generated for Le/Mt as well as Le/Ca by aligning the TOG member sequences to the genome using GMAP (Wu and Watanabe, 2005), then visualizing the correspondences between the physical positions and the genetically derived locations using the CMTV software (Sawkins et al., 2004).

## Results

### TOG sequencing and SNP discovery

**Figure 1:**
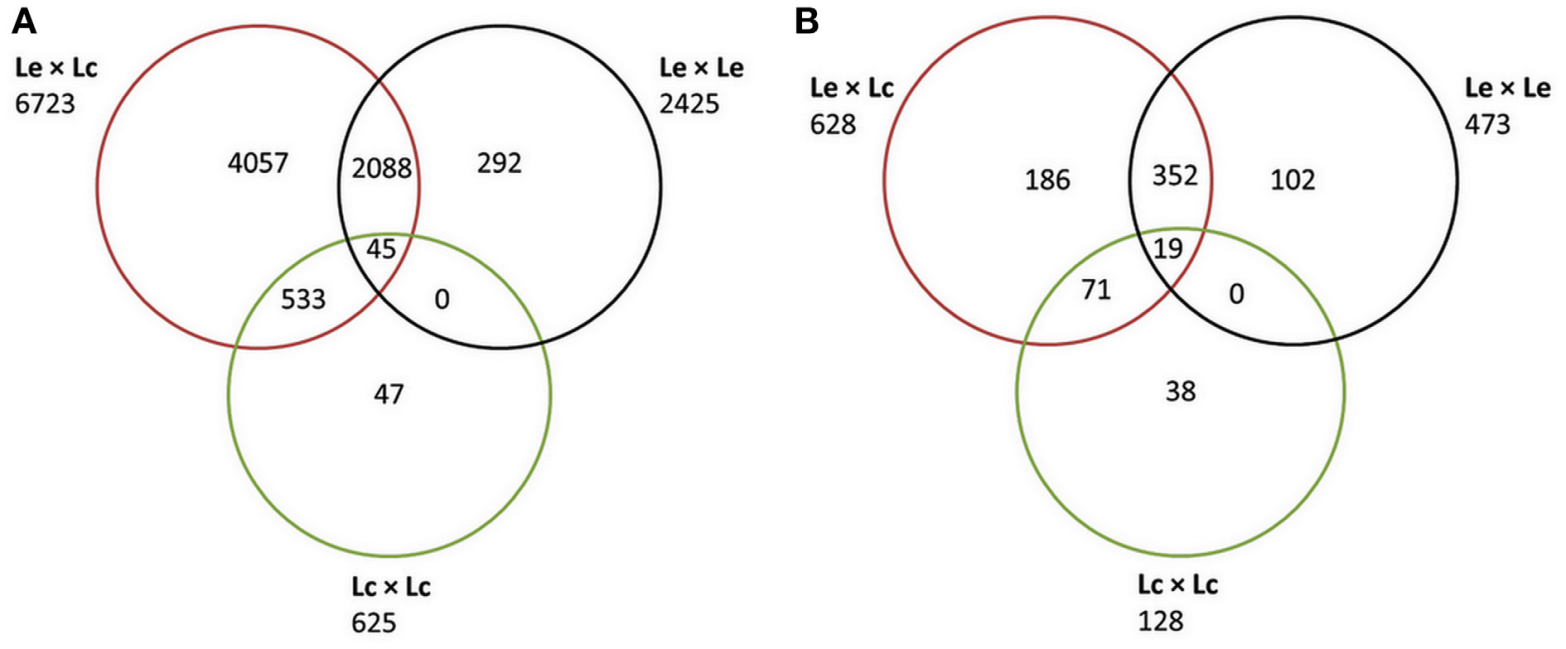
**Venn diagram partitioning out the informativeness of total SNPs between *Lens culinaris* (Lc), between *L. ervoides* (Le), and between the two species (Le and Lc) and their intersections**. **(A)** Classification based on all SNPs discovered using Sanger sequencing of CDC Redberry (Lc), Eston (Lc), L01-827a (Le), and IG 72815 (Le). **(B)** Classification of the subset of SNPs selected for the Le768 Illumina GoldenGate OPA.

Sequence data were obtained for 1107 of 1440 TOG primer sets used, across all four genotypes. A total of 9575 polymorphisms were identified in 1100 unique genes (Additional File 1a). Of these, 2513 were reported as indels or multiple nucleotide polymorphisms (MNPs). The remaining 7062 SNPs were found to be polymorphic in either of the four genotypes and could be present in any of the combinations derived from these genotype (Lc × Lc; Lc × Le; Le × Le; Figure 1A). The largest numbers of SNPs (6723; 9570.2%) were identified between Le and Lc, with fewer (2425; 34.3%) between the two Le wild accessions, and only 625 (8.8%) between the two Lc accessions. Although only 45 SNPs (0.63%) were common to all three comparisons (Lc vs. Le, Le vs. Le, and Lc vs. Lc), many more SNPs (2088; 29.6%) were common to both Le vs. Le and Lc vs. Le combinations (Figure 1A).

### SNP assay development and genotyping

Of the 9575 SNPs submitted to Illumina for Assay Design Tool (ADT) scoring, only 1470 scored higher than 0.5 and were considered for possible inclusion in the OPA design. The 1470 SNPs represented polymorphism in 765 non-redundant genes. For each gene, a single SNP, maximally polymorphic among the four genotypes, was chosen for inclusion in a 768-SNP GoldenGate assay. The majority of SNPs (81.8%) were polymorphic between at least one Lc and at least one Le genotype, 61.6% of the SNPs were polymorphic between the two Le genotypes, and 16.7% were polymorphic between the two Lc genotypes (Figure 1B). The most informative markers were a set of 19 SNPs that were present between the two Lc, the two Le, and at least one Lc and one Le genotype. An additional three SNPs identified in the 765 genes were included to constitute a 768-SNP GoldenGate array for Le (Additional File 1b).

Of the 768 SNPs genotyped via the GoldenGate assay on the LR-66 population, 66 (8.6%) failed and an additional 61 (7.9%) were difficult to score (Additional File 1b). Only 29 marker loci (8%) were severely distorted (*p* < 0.01) and all were skewed toward the Le parent, IG 72815. Of the 31 SNPs from Sharpe et al. (2013) that were genotyped via KASP assays, 20 were polymorphic in the LR-66 population (Additional File 1c). Based on Sanger sequencing data for the Le parents of LR-66, 392 loci were expected to be polymorphic and segregating in the F_2_ population; indeed, 363 loci yielded data amenable for use in linkage mapping (Additional File 1b).

Of the 110 KASP assays designed to monitor SNPs in TOGs, 10 failed to amplify fragments. Of the 100 remaining, 71 were polymorphic when tested on the two Lc genotypes, CDC Redberry and Eston but only 11 of the 71 were segregating in the Lc mapping population (LR-18: CDC Robin × 964a-46) and could be mapped along with additional SNPs (Fedoruk et al., 2013). None of these 11 markers were polymorphic in the Le population LR-66.

### First generation genetic map of *L. ervoides*

**Figure 2:**
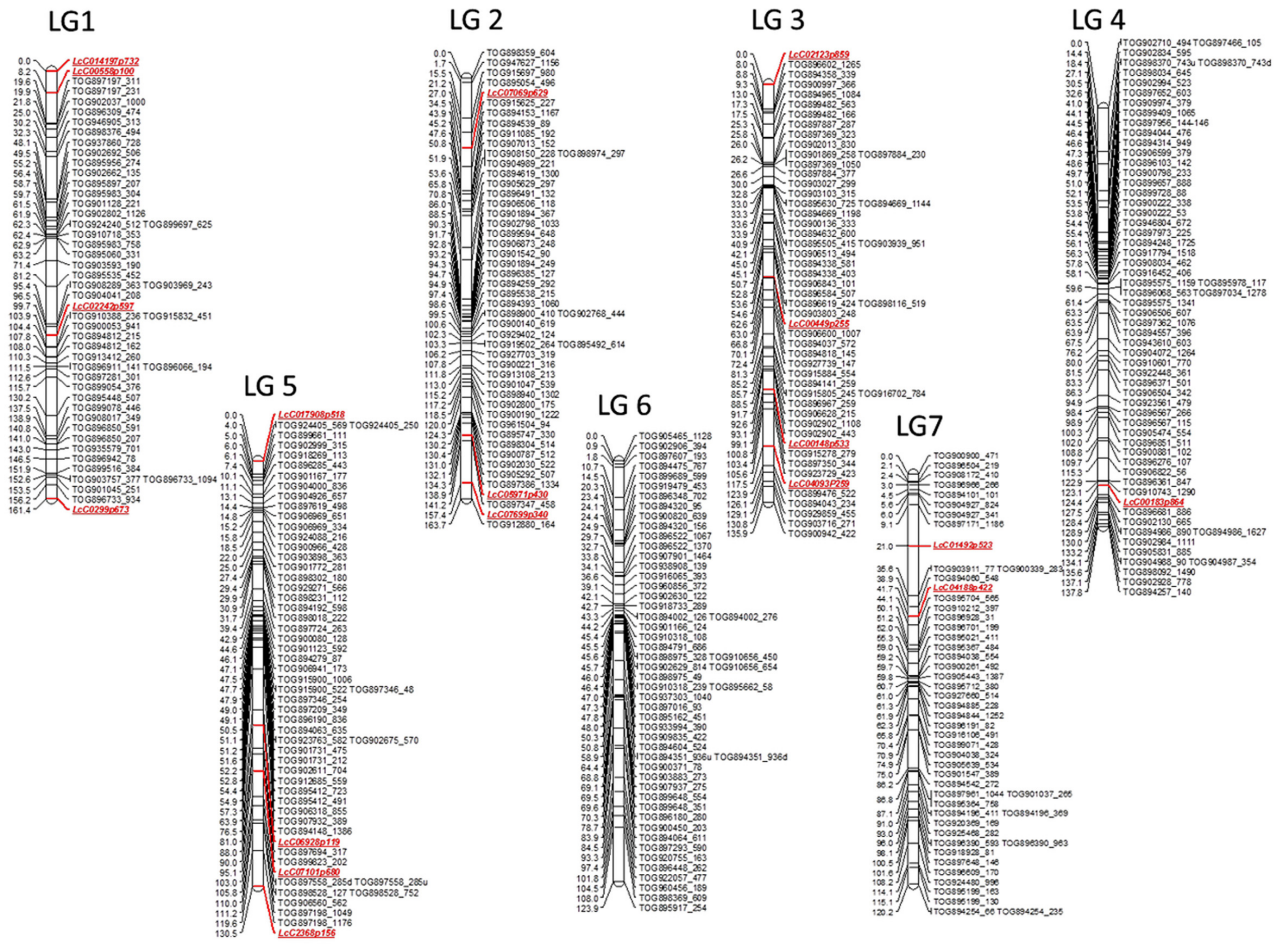
**First generation genetic map of wild lentil species *L. ervoides***. The genetic map based on F_2_ mapping population LR-66 (L01-827a × IG 72815) is comprised of 377 gene based markers including 359 lentil TOGs and 18 other SNP, KASP (LcC) markers. The seven linkage groups likely represent the seven chromosomes of wild lentil and are designated as LeLG1-LeLG7. Map distances (cM) are presented on the left side of the bars and corresponding markers are listed on the right side of the bars. Marker classes are color coded as LeTOGs (black) and LcC (red).

A total of 377 markers mapped into seven linkage groups (LeLG1-LeLG7; Figure 2), which likely represent the seven chromosomes of Le. This genetic map spans 973.7 cM with an average inter-marker distance of 2.6 cM. Similar numbers of marker loci mapped on to each linkage group, varying from 50 (LeLG1 and LeLG7) to 62 (LeLG4). The linkage groups were numbered to best match those in the Lc map of Sharpe et al. (2013).

### Comparative mapping with closely related legumes

**Figure 3:**
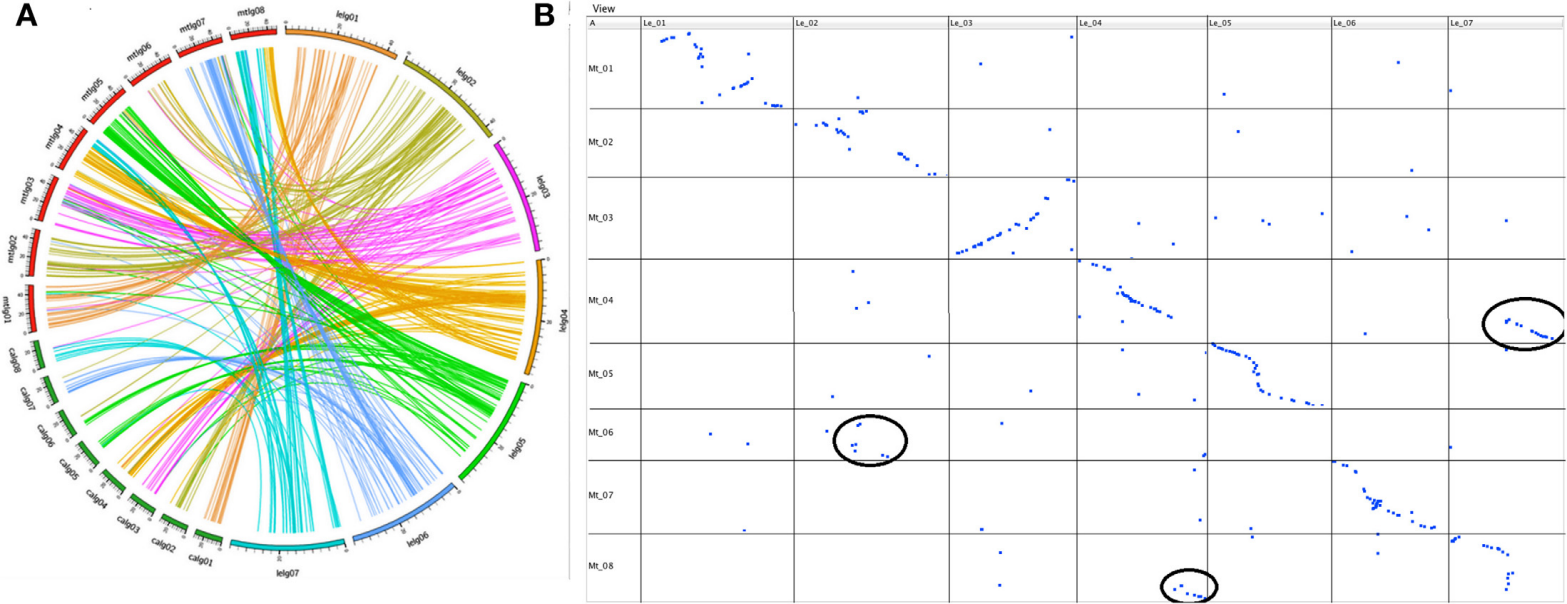
**(A)**. Syntenic relationship of wild lentil species *L. ervoides* with two sequenced legume species [*M. truncatula* (mtlg01-08) and chickpea (*C. arietinum*; calg01-08)] shown by comparing the map position of 377 Le TOGs markers with corresponding map positions in other two legume species. **(B)** Dot plot representing correspondences between *L.ervoides* linkage groups LeLG1 to LeLG7 (top) and *M. truncatula* linkage groups MtLG1 to MtLG8 (left side) based on alignment and visualization using GMAP and CMTV software. Major translocations are circled in black.

Of the TOGs that mapped on the seven linkage groups of Le, 315 (99%) had orthologs located on the eight chromosomes of Mt and 128 of these had orthologs that also mapped to the eight chromosomes of chickpea (Additional file 2). When the maps of the three species were aligned, extensive stretches of shared collinearity among them were evident (Figure 3; Additional file 3). A minimum of 41 (LeLG1) and a maximum of 56 (LeLG4) TOGs per Le linkage group had corresponding loci mapped in Mt (Additional file 2). One of the TOGs (TOG963130) was on a Mt scaffold that had not yet been incorporated into a Mt chromosome but mapped on Le-LG4 at 44.5 cM. A closer look at the comparison between Le and Mt revealed that Mt Chromosome 6 is collinear with the middle of LeLG2 (Figure 3B). There is a major translocation involving Mt chromosomes 4 and 8 relative to LeLG4 and LeLG7, and major inversions within LeLG1 relative to Mt chromosome 1 and within LeLG3 relative to Mt chromosome 3 were also evident.

The 128 TOGs mapped in Ca, Mt and Le, covered all Ca chromosomes with 8–22 TOGs per chromosome and corresponded to loci on all 7 Le linkage groups with 9–21 TOGs per linkage group (Additional file 2). As was seen with the Le-Mt comparison, there were large stretches of conserved synteny between Le and Ca (Additional file 3). The additional chromosome in Ca appears to result from a splitting into chromosomes 5 and 6 of the ancestral chromosome that corresponds to LeLG5 into Ca.

### Identifying translocations in *L. ervoides* vs. *L. culinaris*

**Figure 4:**
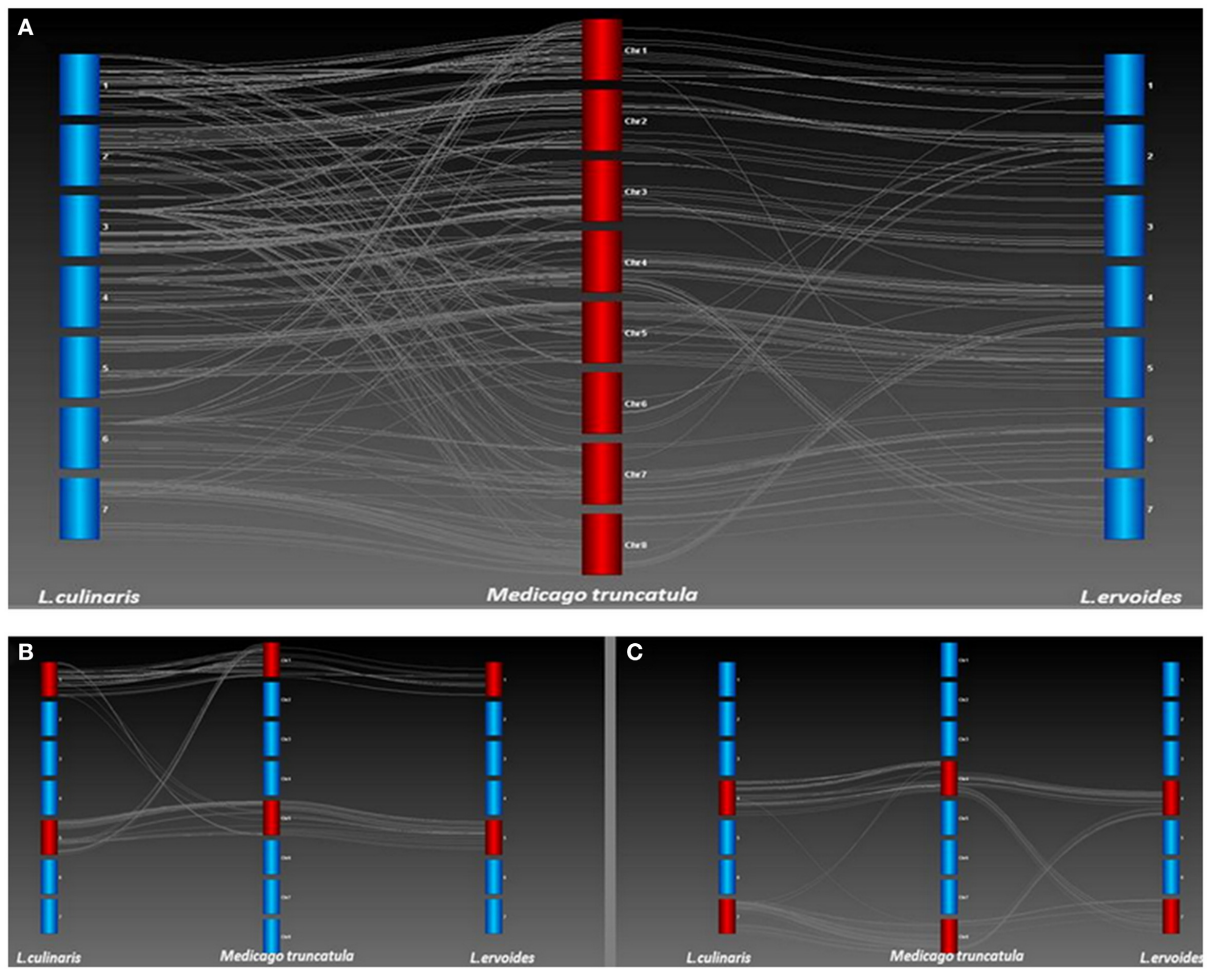
**(A)** Collinearity between *L. culinaris* (Lc) and *L. ervoides* (Le) with respect to *M. truncatula* (Mt). Genetic maps of Lc and Le were compared with the Mt 3.5.1 genome build (Lavin et al., 2005) to identify chromosomal re-arrangements. Each bar represents a linkage group of Lc or Le or chromosome of Mt. Lines between linkage groups and Mt chromosomes represent Homologs as determined through a BLAST of the *Lens* sequences against the Mt genome. **(B)** A translocation was identified on LG1 and LG5 of Lc with respect to Mt chromosomes 1 and 5 but not observed in Le LR-66. **(C)** A translocation was identified on LG4 and LG7 of Le with respect to chromosomes 4 and 8 of Mt but not observed in Lc LR-18.

A high degree of collinearity was observed for the two lentil species in comparison with Mt, but a few key differences were also apparent (Figure 4A). Most notably, the break in conserved synteny observed on LcLG1 and LcLG5 with respect to Mt chromosomes 1 and 5 (Sharpe et al., 2013) was not observed in Le (Figure 4B). A translocation that occurs in Le with segments of LeLG4 and LeLG7 with respect to Mt chromosomes 4 and 8 is not as apparent in the LR-18 Lc linkage map (Sharpe et al., 2013; Figure 4C). In a different Lc mapping population (LR-03) derived from the cross ILL 1704 × ILL 7537, however, additional markers mapped on LcLG4 and LcLG7 (unpublished data). These are monomorphic in LR-18 but homologous to Mt chromosomes 8 and 4, respectively, suggesting the same translocation with respect to Mt occurs in both *Lens* species.

**Figure 5:**
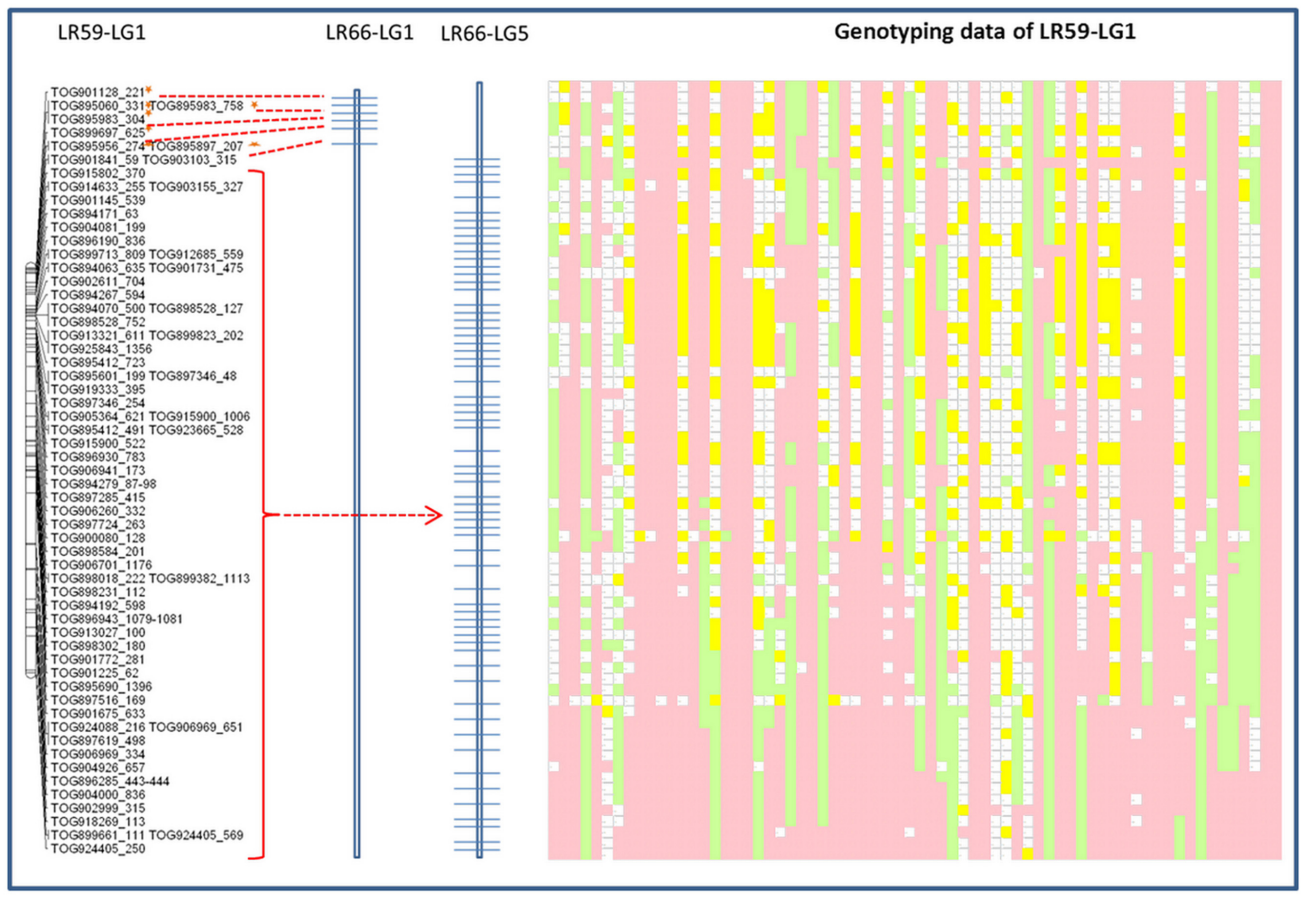
**Schematic representation of the distribution of Le768 OPA assay TOGs mapped in LG1 in the interspecific LR-59 population**. Common markers mapped in the LR-66 population are represented on LR-66-LG1 and LR-66-LG5. Segregation of the same common markers in LR-59 is depicted by genotyping data. Each column represents an individual RIL and each row represents a locus genotyped using the TOGs. Pink blocks represent Eston (Lc) alleles, green blocks represent L01-827a (Le) alleles, yellow blocks are heterozygous loci, and blank blocks represent missing data.

Genotypic data from one of the linkage groups from the Lc × Le interspecies introgression population, LR-59, were significantly distorted (69% skewed) toward the Lc parent alleles and aligned with portions of both LeLG1 and LeLG5 (Figure 5). Disproportionate numbers of heterozygous loci were evident in the region surrounding the break in collinearity in this linkage group. Segregation distortion or breaks in collinearity were not observed in the LR-59 linkage groups that correspond to LeLG4 and LeLG7 (data not shown).

## Discussion

### SNP discovery and genotyping

Until recently, microsatellites and amplified fragment length polymorphisms (AFLP) were the marker systems of choice for lentil molecular marker based linkage studies. With the advent of high-throughput sequence technology, thousands of SNPs have now been identified in lentil germplasm (Kaur et al., 2011, 2013; Sharpe et al., 2013) and new, SNP-based molecular markers have been developed (Kaur et al., 2013; Sharpe et al., 2013). These high-throughput SNP genotyping platforms are useful for rapidly genotyping mapping populations and germplasm. Several SNP genotyping methods have been developed, such as array methodologies (Sapolsky et al., 1999; Ishikawa et al., 2005), pyrosequencing (Ronaghi et al., 1998; Ahmadian et al., 2000; Oliphant et al., 2002), and other high throughput techniques. Illumina GoldenGate assays have been used extensively because of their high throughput nature, multiplexing capability, accuracy, speed, and low cost per data point compared to other genotyping platforms. This genotyping platform is suitable for large-scale SNP genotyping but is not economical for small numbers of SNPs. KBioscience (now LCG Genomics) developed KASP assays to target SNPs and indels. These assays are simple and cost-effective, and are well adapted to low- to medium-throughput genotyping projects (Chen et al., 2010).

SNPs identified in this study originated from Sanger sequencing of a set of legume TOGs in two Le (wild lentil) and two Lc (cultivated lentil) accessions. These SNPs were either polymorphic in cultivated lentil (Lc-Eston vs. Lc-Reberry), wild lentil (Le-L01-827a vs. Le-IG72815) or cultivated vs. wild (Lc vs. Le; Eston/L01-827a, Eston/IG72815, Redberry/L01-827a, Redberry/IG72815). As expected, due to the occurrence of the same SNP in multiple groups, the total number of the SNPs from each group has exceeded the final SNPs (9575; Figure 1A). The majority of the SNPs were between Le and Lc, in part because there were twice as many possible comparisons but also because these are divergent species. Many more SNPs were observed between the two wild Le accessions and far fewer were observed between the two cultivated Lc accessions. Although the two Lc accessions represent the two major commercial classes of lentils (small red and small green) grown in Canada, they are both adapted to western Canadian growing conditions and likely do not represent the maximum genetic diversity that exists in the Lc genepool.

Due to the occurrence of more polymorphisms in Le and between the wild and cultivated species, an Illumina GoldenGate OPA was more practical for linkage mapping in wild lentil and interspecies hybrid populations, but not for linkage mapping within cultivated Lc. The Le768 OPA was used successfully to genotype an F_2_ mapping population, LR-66, derived from a Le × Le cross. Only 8.6% of the loci failed, which is consistent with other experiences with unvalidated OPAs (Rostoks et al., 2006; Hyten et al., 2008). The OPA was not used to genotype the Lc × Lc mapping population as the majority of the loci were expected to be monomorphic.

In LR-66, a Le × Le F_2_ population, 392 loci were anticipated to be polymorphic based on the Sanger sequencing results of parental lines, and we obtained high quality genotypic data for 363 loci. The reduced number of segregating loci were explained by parental genotypes at 29 loci (8.0%) that did not match with the Sanger sequence genotypes for the two accessions and a further 20 loci genotyped as heterozygous in one or other of the mapping parents but not reported as such in the Sanger data. Heterogeneity of parental seed stocks or residual heterozygosity in plants used for DNA sequencing vs. those used to develop the LR-66 population may underlie such genotypic differences. Another problem encountered with 61 loci, caused by trying to map an F_2_ population, was the difficulty in distinguishing heterozygotes from homozygotes. Genotypes for these loci were not included in the mapping data.

### First generation of an intra-specific map of wild lentil

This study resulted in the generation of the first intra-specific gene-based map of wild lentil, established using a population of F_2_ individuals of a cross between two wild Le accessions, L01-827a and IG 72815. The map consists of seven linkage groups, which likely represent the seven chromosomes of Le. Many of the early lentil maps were constructed using populations derived from inter-specific crosses between cultivated lentil and a wild parent from the secondary gene pool (Havey and Muehlbauer, 1989a,b; Tahir et al., 1993; Eujayl et al., 1998) to generate sufficient numbers of polymorphic markers. The recent development of improved marker technologies has enabled mapping with intra-specific crosses within the primary gene pool (Phan et al., 2006; Tullu et al., 2006, 2008; Fedoruk et al., 2013; Sharpe et al., 2013). To date, no maps of any of the wild lentil species have been published.

An important feature of existing lentil genetic maps is the clustering of markers in the middle, upper-middle, or lower-middle part of linkage groups indicative of reduced recombination in these regions (Hamwieh et al., 2005; Gupta et al., 2012). Similar clusters of markers were observed in the Le map in the current study, and in the recent SNP linkage map of cultivated lentil (Sharpe et al., 2013) containing more markers, both of which use gene-based markers. Such regions may span the location of the centromeres, which are often gene-poor and exhibit suppressed recombination.

The Le map from the current study consists of 377 gene-based markers of which 359 are TOGs that have been mapped in at least two other legume species. Apart from the model legumes with fully sequenced genomes, these markers have also been mapped in chickpea (Hiremath et al., 2012), common bean (Blair et al., 2013) and pigeon pea (Saxena et al., 2012), which should improve the ability to make cross-generic comparisons.

### Comparative mapping with closely related legumes species

Through comparative mapping, predicting gene order and gene content across members of the same family is possible (Tanksley et al., 1992; Livingstone et al., 1999; Wilson et al., 1999). Lentil has been compared to Mt and pea using isozymes (Weeden et al., 1992) and large blocks of conserved synteny were demonstrated. Phan et al. (2006), Alo et al. (2011), and Gupta et al. (2012) use gene-based SSRs to compare Mt and Lc. Sharpe et al. (2013) confirm the extensive level of collinearity between Mt and Lc using gene-based SNP markers. The availability of single copy conserved sequences and markers for orthologous genes from heterologous legume species increases the reliability of comparative maps. Legume TOG markers have been used to study evolution across pigeon pea (Kassa et al., 2012), common bean (Blair et al., 2013) and other legumes (Choi et al., 2004a,b). The set of TOGs used in this study allows these comparisons to extend to wild lentil as well.

### Conserved synteny between *L. ervoides* and *M. truncatula* and *C. arietinum*

Several research groups have compared the genome structure of Mt and various crop legumes (Choi et al., 2004a,b; Zhu et al., 2005). Lentil and Mt, its most closely related model legume species, diverged some 24 (Lavin et al., 2005) to 38 (Sharpe et al., 2013) million years ago. By using the TOG markers, a comparative analysis was carried out to demonstrate a high level of conserved macrosynteny between Le and Mt (Figure 3B). With the exception of Mt chromosome 6, extensive collinearity between the two species was evident. Mt chromosome 6 is unusual in that it has very few genes and appears to have originated as a fragment of a larger chromosome of the ancestral legume (Kalo et al., 2004; Cannon et al., 2006; Young et al., 2011). This Mt chromosome appears to have broken away from the ancestor of linkage group 2 in both Le (Figures 3B, 4A) and Lc (Sharpe et al., 2013) and chickpea (Additional file 3).

A large inversion in the middle of LeLG1 relative to Mt chromosome 1 is also found in Lc (Figure 4B). The only other notable exception to this collinearity is a translocation involving Mt chromosomes 4 and 8 relative to LeLG4 and LeLG7. Kamphuis et al. (2007) provide strong evidence of a reciprocal translocation in the sequenced Mt reference genotype A17 involving chromosomes 4 and 8 that is not present in other Mt accessions. This suggests the break in collinearity seen in Le relative to A17 is a specific consequence of chromosomal rearrangements in genotype A17, rather than that characteristic of species Mt or in the common ancestor of *Medicago* and *Lens*.

A set of the legume TOGs was identified and mapped in chickpea (Hiremath et al., 2012), allowing a comparison of common markers in all three genomes (Figure 3; Additional file 3). All 128 TOGs mapped in all three species and shows a high level of conservation of synteny among the species and can also be used as a resource to identify syntenic regions in other species. Clear blocks of shared synteny cover all linkage groups of the three species and key chromosomal rearrangements distinguish them. The identification of conserved regions relative to these fully sequenced legumes will aid more detailed study of legume genome evolution and the comparative analysis of the genic basis of traits. It is now also possible to use comparative information from these better-characterized species to accelerate candidate gene identification in lentil.

### Ancient translocation in the genus *Lens*

New species arise when plants are reproductively isolated. Over time, chromosomal rearrangements preclude them from being inter-fertile when reintroduced to a common environment. Le and Lc diverged less than a million years ago (Sharpe et al., 2013) but have already undergone chromosomal rearrangements that make the production of fully fertile offspring difficult (Tadmor et al., 1987; Weeden et al., 1992).

The gene-based linkage map of Le was compared with a map of Lc (Fedoruk et al., 2013) using Mt as an intermediate, a necessary step because there were few polymorphic TOGs in Lc. Even fewer polymorphisms could be mapped in the Lc mapping population LR-18, resulting in few common markers between the two species. Because the Le map consisted of gene-based TOG and SNP markers, identifying orthologs in the fully sequenced Mt genome was possible. Comparative mapping showed a direct and simple relationship between Le linkage groups and Mt chromosomes. For Lc, however, the comparison appeared more complex but this is likely a result of the comparison being less robust due to the source of the SNP markers used in that map. Those SNPs were derived from 3′ end sequencing of transcripts (Sharpe et al., 2013) whose divergent nature makes comparisons to Mt difficult. In addition, the limited dataset available for Lc means some of the Homologs identified using reciprocal BLAST could have been paralogs, leading to less well-defined collinearity (Figure 4A). Despite this, it was still possible to identify gross level rearrangements that define the two species.

Previous studies in Lc and Le established the foundation of a single ancient translocation that separates the two species (Ladizinsky et al., 1985; Tadmor et al., 1987; Weeden et al., 1992). The reciprocal translocation observed on linkage groups 1 and 5 of Lc with respect to Mt (Sharpe et al., 2013; Figure 4B) is not evident in Le and likely represents this key difference between the two *Lens* species. The cotyledon color gene, *Yc*, maps to LcLG1 (Fedoruk et al., 2013) near to the breakpoint of this translocation. A similar observation was made by Weeden et al. (1992) based on mapping of interspecific hybrids between Lc and Le. They identify translocation break point using morphological (*Yc*) and isozyme markers (Pgm-2, Aco-1). The first 5 cM of LcLG1 have Homologs from Mt chromosome 5. There is a major gap of 16.7 cM in this region in Lc, which likely reflects a loss of chromosomal segment or re-arrangement in the Lc genome. Similarly for LcLG5, a group of markers that mapped between 64.4 cM and 100.2 cM corresponds to Homologs from Mt chromosome 1. No such translocation was evident in Le relative to Mt (Figure 4B), suggesting this is unique to Lc.

The presence of a translocation in Lc that is not present in Le is corroborated by segregation of TOG loci in an interspecific RIL population, LR-59, derived from an Lc × Le cross. In this population, markers at the top of one LR-59 linkage group correspond to LeLG1 while the rest of the linkage group corresponds to markers mapping to LeLG5 (Figure 5). Many of the markers on this LR-59 linkage group were distorted toward the Lc parent alleles, suggesting preference for the Lc structure during meiotic events that occurred during the development of the inbred lines. Heterozygous genotypes that are atypical for RILs, were observed in the region surrounding the putative break point, suggesting incomplete pairing and recombination during meiosis as would happen in the case of mis-matched chromosomes. Segregation distortion and unequal crossing over as well as the presence of transpositional elements have been associated with chromosomal rearrangements in interspecific *Lens* hybrids (Galasso, 2003) and confirmed here for Le and Lc.

The translocation observed between Le and Mt involving Mt chromosomes 4 and 8 is absent in Lc based on the LR-18 map (Figure 4C). This suggests the presence of a second translocation distinguishing Le from Lc which had not been observed previously. Genotypic data from these regions in an additional Lc mapping population, however, indicated the presence of additional markers, monomorphic in LR-18, that fall in the region spanning the translocation in Mt and confirm that Lc and Le share the same structure with no evidence in our data of additional translocations. Genotypic data from the LR-59 interspecific population corresponding to these two linkage groups were not distorted toward one or other parental allele and were completely collinear with LeLG 4 and LeLG 7, further confirming the lack of translocation in this region within Lc relative to Le. The lack of a translocation further confirms the observations of Kamphuis et al. (2007) that A17 has a unique translocation and suggests that the common ancestor to both Mt and *Lens* share similar structure in these two chromosomes.

## Summary

With the exception of this one major translocation, the other five linkage groups appear to be collinear, suggesting interspecies hybrids could be used to effectively introgress genes from wild lentil into cultivated lentil unless they occur in or near the translocation breakpoint. Mapping of genes or traits of interest in LR-66 or other Le populations will allow for the development of markers that can be used to track introgression following crossing into cultivated lentil and facilitate selection for individuals carrying the Le alleles in the regions of interest. The construction of a genetic linkage map of the wild lentil *L. ervoides* and the identification of the chromosomal changes that differentiate it from cultivated lentil provide lentil geneticists a bridge to the genomic information and the genetic resources of better-characterized legumes. Knowledge of the colinearity among these legume species will allow us to leverage information from fully sequenced species for molecular marker-based breeding and discovery of genetic basis of traits in *Lens* species.

## Author contributions

Kirstin E. Bett, Albert Vandenberg, and Douglas R. Cook were PIs on the projects that led to this manuscript. Douglas R. Cook, R. Varma Penmetsa, Noelia Carrasquilla-Garcia, and Andrew D. Farmer developed the legume COS markers. Andrew D. Farmer developed the bioinformatic pipelines. Albert Vandenberg selected the genotypes for sequencing and Sally L. Vail developed the LR-66 mapping population. Sally L. Vail, Noelia Carrasquilla-Garcia, and R. Varma Penmetsa carried out the initial sequencing of the four Lens genotypes. Kirstin E. Bett, Andrew D. Farmer, and R. Varma Penmetsa designed the GoldenGate OPA. Kirstin E. Bett analyzed the GoldenGate data and did the genome mapping with Neha Gujaria-Verma. Andrew D. Farmer, Kirstin E. Bett, and Neha Gujaria-Verma did the cross-species comparisons. Neha Gujaria-Verma and Kirstin E. Bett wrote the first draft of the manuscript.

## Funding

The authors acknowledge funding from the Saskatchewan Pulse Growers (BRE0820) to Kirstin E. Bett, Albert Vandenberg, and Sally L. Vail and from the US National Science Foundation DBI 0605251 to Douglas R. Cook.

### Conflict of interest statement

The Associate Editor Paul Gepts declares that, despite being affiliated to the same institution as authors Sally L. Vail, Noelia Carrasquilla-Garcia, R. Varma Penmetsa and Douglas R. Cook, the review process was handled objectively and no conflict of interest exists. The authors declare that the experiments of this study comply with the current laws. We confirm to have the authority to publish this work and that the manuscript has not been published before and is not under consideration for publication elsewhere. The authors declare that the research was conducted in the absence of any commercial or financial relationships that could be construed as a potential conflict of interest.

## Supplementary material

All contigs and identified SNPs are available through the KnowPulse webportal at http://knowpulse2.usask.ca/portal/node/53 and in Additional file 1B.
