# Supplementary figures and images for: Genetic mapping of legume orthologs reveals high conservation of synteny between lentil species and the sequenced genomes of Medicago and chickpea
Source: Front Plant Sci. 2014 Dec 5;5:676. doi: 10.3389/fpls.2014.00676 (PMC4256995; doi:10.3389/fpls.2014.00676)

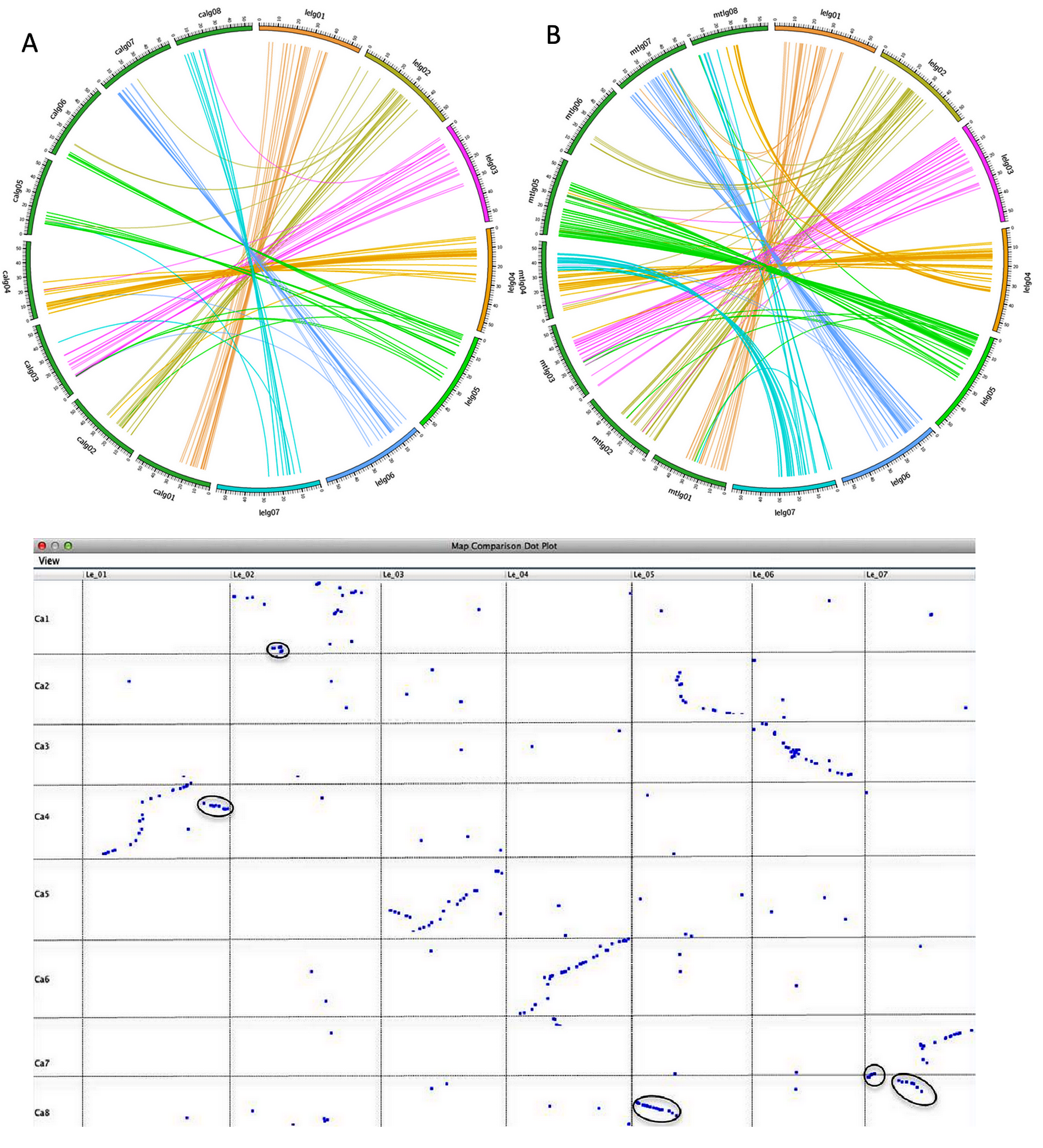

Supplement: Supplementary file 3 [file Image1.TIF]
